# Supplementary material for: Neural Correlates of the Shamanic State of Consciousness
Source: Front Hum Neurosci. 2021 Mar 18;15:610466. doi: 10.3389/fnhum.2021.610466 (PMC8012721; doi:10.3389/fnhum.2021.610466)
Supplement: Supplementary file 1 [file Data_Sheet_1.PDF]

Supplemental Table 1. Demographics of shamanic practitioners and controls included in analysis.

	Shamanic Practitioners (n = 18	Controls (n = 19)
**Age (median)**	56 (5.5)	57(12)
**Sex (% male)**	50%	47%
**Years of Practice**	19.5(21.125)	---
**Healing Sessions Per Month**	12.75(14.25)	---

Supplemental Table 2. Statistical comparison of OAV domain scores between shamanic practitioners and healthy individuals under the influence of psychedelics (Studerus et al., 2010).

	Ketamine (df = 178)			Psilocybin (df = 343)			MDMA (df = 118)		
**OAV Domain**	*t*	*p*	95% CI	*t*	*p*	95% CI	*t*	*p*	95% CI
Complex imagery	**4**	**<.001**	**[14.57, 42.88]**	**2.68**	**.0077**	**[5.54, 36.09]**	**7.95**	**<.001**	**[34.86, 58.002]**
Exp. of unity	**2.83**	**.0052**	**[6.27, 35.12]**	**3.8**	**<.001**	**[13.05, 41.06]**	**4.11**	**<.001**	**[15.64, 44.66]**
Spiritual exp.	**6.43**	**<.001**	**[26.17, 49.33]**	**6.72**	**<.001**	**[27.65, 50.55]**	**9.34**	**<.001**	**[36.59, 56.27]**
Blissful state	**4.51**	**<.001**	**[16.75, 42.82]**	**2.93**	**.0036**	**[6.66, 33.91]**	1.5	.14	[-3.6, 25.98]
Disembodiment	.737	.46	[-9.7, 21.26]	**3.96**	**<.001**	**[13.74, 40.90]**	**4.37**	**<.001**	**[16.28, 43.32]**
Insightfulness	**4.83**	**<.001**	**[17.3, 41.21]**	**4.44**	**<.001**	**[15.23, 39.41]**	**6.19**	**<.001**	**[25.03, 48.57]**
Elem. visual alt.	1.1	.28	[-6.64, 23.21]	1.11	.27	[-24.77, 6.91]	**7.35**	**<.001**	**[25.28, 43.93]**
Changed percepts	.099	.92	[-12.88, 14.25]	.64	.52	[-19.62, 9.99]	0.48	.63	[-11.52, 18.94]
Audio-visual syn.	**2.17**	**.031**	**[-32.94, -1.57]**	**3.16**	**.0017**	**[-42.45, -9.85]**	0.045	.96	[-10.22, 9.77]
Imp. cont. & cogn.	**3.88**	**<.001**	**[-35.8, -11.65]**	**2.86**	**.0045**	**[-24.62, -4.55]**	1.41	.16	[-16.91, 2.86]
Anxiety	**2.1**	**.037**	**[-17.82, -0.56]**	1.12	.27	[-12.96, 3.57]	.012	.99	[-5.39, 5.33]

df = degrees of freedom; *t* = test statistic; *p* = significance; CI = confidence interval [lower, upper]

Supplemental Table 3. Written responses detailing religious and spiritual traditions incorporated into training and/or practice of shamanic practitioners.

Shamanic Practitioner ID	Religious/Spiritual Traditions Cited in Training/Practice
4	Q'ero cosmology (Peruvian)
8	Esoteric Christianity, Cabala, Native American, Druidic
100	My trainings have not had any religious or spiritual traditions. However in my own practice I have connected with deities and angels from various religions, both current and ancient.
101	Peruvian, Northern European, Slavic, Native American, Haitian Vodou
104	None
108	Tibet, Q'ero cosmology (Peruvian)
115	Inca, Celtic, Mapuche, Siberian traditions. The Northern Peruvian techniques often are infused with Catholic practices.
116	Christianity, Islam, various cultural mythologies (usually Greek)
125	Core shamanism, Hawaiian shamanism
127	Cherokee, Lakota, Peruvian, Santeria, Celtic, Hindu, Buddhist, Christian
128	None, I have modified all my trainings to make them work better for me. For instance, I have added angelic work into my shaman work
133	I am a member of the Choctaw Nation
136	Michael Newton's work as my own personal cosmology
137	Inca and Amazon
139	Q'ero cosmology (Peruvian)
141	Native American, Nepalese & Tibetan shamanism, South American indigenous
145	None
148	My teachers were mainly Cherokee, Navajo, Lakota, Mayan and some Incan
